# Reviewed article: Silva GA, Santos CH, Leão-Cordeiro JAB, Silva AMTC. Temporal trends and regional disparities in mortality from acute myocardial infarction in Brazil: a time series study, 2015-2024. Epidemiol Serv Saude. 2026:35;e20260533

**DOI:** 10.1590/S2237-96222026v35e20260533.b

**Published:** 2026-09-25

**Authors:** Luciana Bertoldi Nucci

**Affiliations:** 1 Pontifícia Universidade Católica de Campinas, Campinas, SP, Brazil Pontifícia Universidade Católica de Campinas Campinas SP Brazil

## First round comments

O manuscrito descreve a tendência temporal da mortalidade por infarto agudo do miocárdio no Brasil, tema de interesse para a área. No entanto, aspectos metodológicos e de apresentação dos resultados requerem atenção antes que o trabalho possa ser considerado para publicação. As recomendações a seguir visam aprimorar a qualidade e o rigor do estudo.

1. No resumo é citado que “A tendência temporal foi estimada por regressão log-linear e método de Prais-Winsten.”, diferente do que está descrito no corpo do texto. Recomenda-se que os autores revisem e harmonizem essas informações, garantindo clareza e precisão na descrição dos procedimentos adotados em todas as seções do manuscrito.

2. A avaliação de tendência temporal deve ser complementada com a estimativa da taxa de variação anual (Annual Percent Change - APC) e seu respectivo intervalo de confiança de 95%. Essa informação é fundamental para que o leitor possa interpretar a magnitude e a direção das tendências observadas com o rigor estatístico adequado. Recomenda-se que essa análise seja apresentada para todas as categorias avaliadas, não apenas para a taxa padronizada por idade.

3. Os autores descrevem o cálculo das taxas padronizadas por dois padrões de referência (mundial e brasileiro), porém não exploram nem interpretam as diferenças observadas entre elas. A comparação entre taxas padronizadas por diferentes populações de referência é, em si, uma informação analítica relevante: reflete as diferenças na estrutura etária dos padrões adotados e, consequentemente, o quanto a composição demográfica local influencia as taxas observadas. Adicionalmente, a magnitude da diferença entre as taxas brutas e as padronizadas merece discussão, uma vez que ilustra o efeito da estrutura etária da população brasileira sobre as taxas observadas e tem implicações diretas para a interpretação dos achados. Recomenda-se que os autores discutam essas diferenças, justifiquem a escolha dos padrões utilizados e contextualizem os resultados em relação ao perfil demográfico da população estudada.

4. Recomenda-se que as taxas padronizadas por idade sejam calculadas para cada região do Brasil, utilizando a população total do país como referência comum. Essa abordagem permitiria isolar o efeito da estrutura etária regional das diferenças observadas nas taxas de mortalidade, qualificando a interpretação dos achados.

5. A ausência de comparação com dados de outras regiões ou países limita a capacidade do estudo de contextualizar os achados e avaliar a magnitude da mortalidade observada em perspectiva mais ampla. Estudos descritivos de tendência temporal contribuem com o conhecimento epidemiológico e, para que cumpram esse papel, devem agregar interpretação além da mera descrição dos dados. Recomenda-se que os autores ampliem a discussão, situando os resultados no contexto da literatura nacional e internacional.

6. Os gráficos, embora ilustrativos, não permitem a identificação dos valores exatos das taxas de mortalidade. Sugere-se que os dados sejam complementados por tabelas suplementares, substituídos por tabela, ou que os valores sejam incorporados diretamente nas figuras, de modo a tornar os resultados mais acessíveis e precisos. Por exemplo, as figuras 2 e 3 poderiam ser descritas em uma tabela, com a descrição de outras variáveis, como sexo, escolaridade, local de ocorrência, incluindo a APC e IC95% de cada categoria. 

Recommendation: Major revision

